# Supplementary material for: Identification of Powdery Mildew Resistance-Related Genes in Butternut Squash (Cucurbita moschata)
Source: Int J Mol Sci. 2024 Oct 10;25(20):10896. doi: 10.3390/ijms252010896 (PMC11507584; doi:10.3390/ijms252010896)
Supplement: Supplementary file 1 [file ijms-25-10896-s001.zip › Supplementary Figures.pdf]

Supplementary Figures

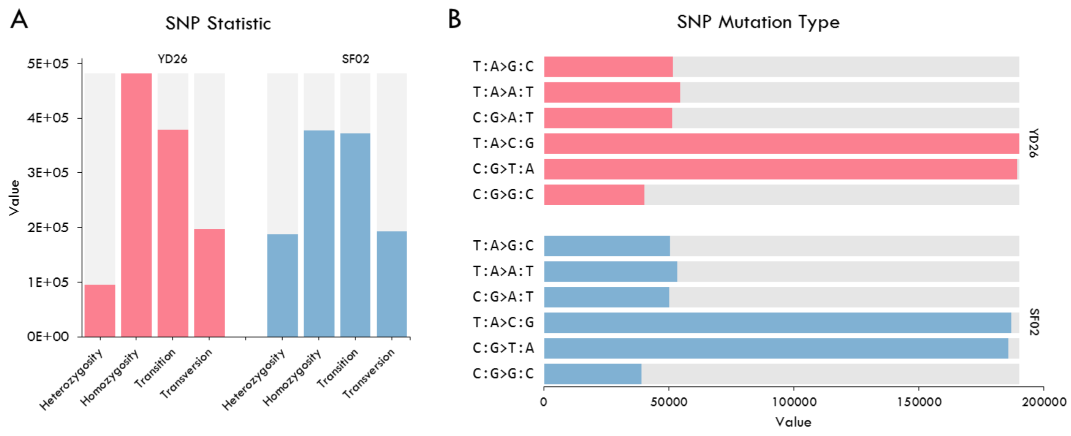

Figure S1. (A) Statistical analysis of SNP mutations in genotypes YD26 and SF02. (B) SNPs mutation changes of YD26 and SF02.

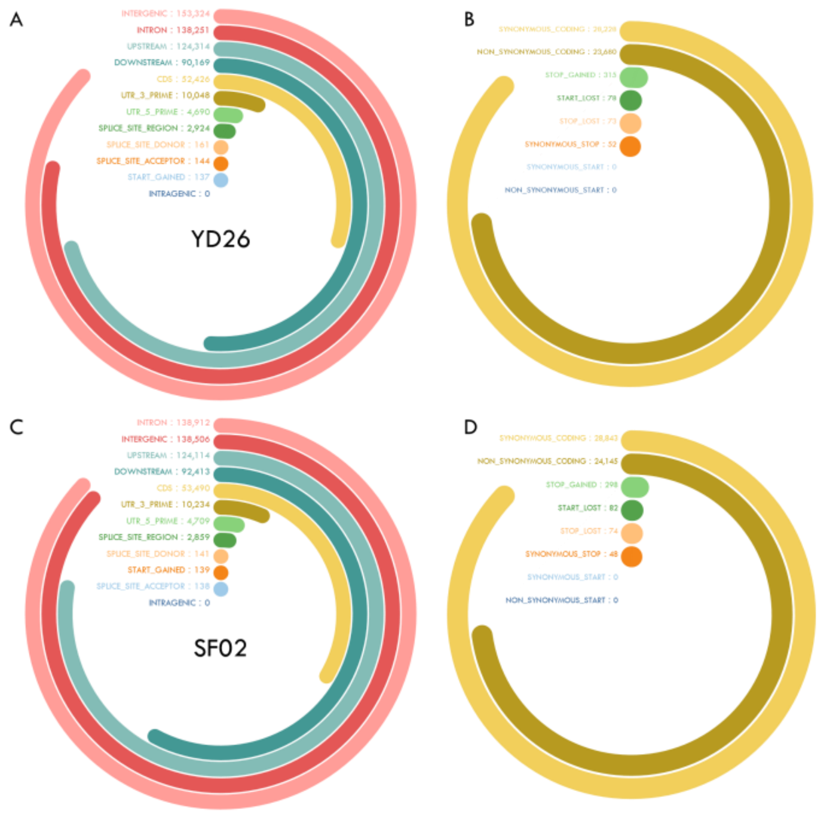

Figure S2: (A) Genome-wide variants in the CDS region of YD26. (B) Annotation of variants and prediction of their effects on genes in YD26. (C) Genome-wide variants in the CDS region of SF02. (D) Annotation of variants and prediction of their effects on genes in SF02.

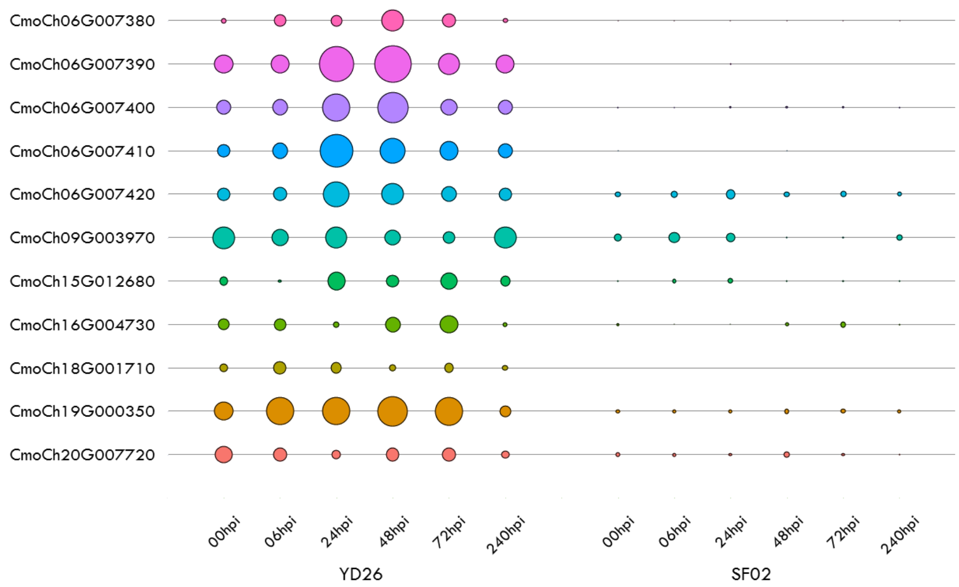

Figure S3 The FPKM values of 11 DEGs consistently up-regulated across all time points in YD26 compared to SF02. Bubble sizes correspond to the FPKM values, indicating the level of gene expression.

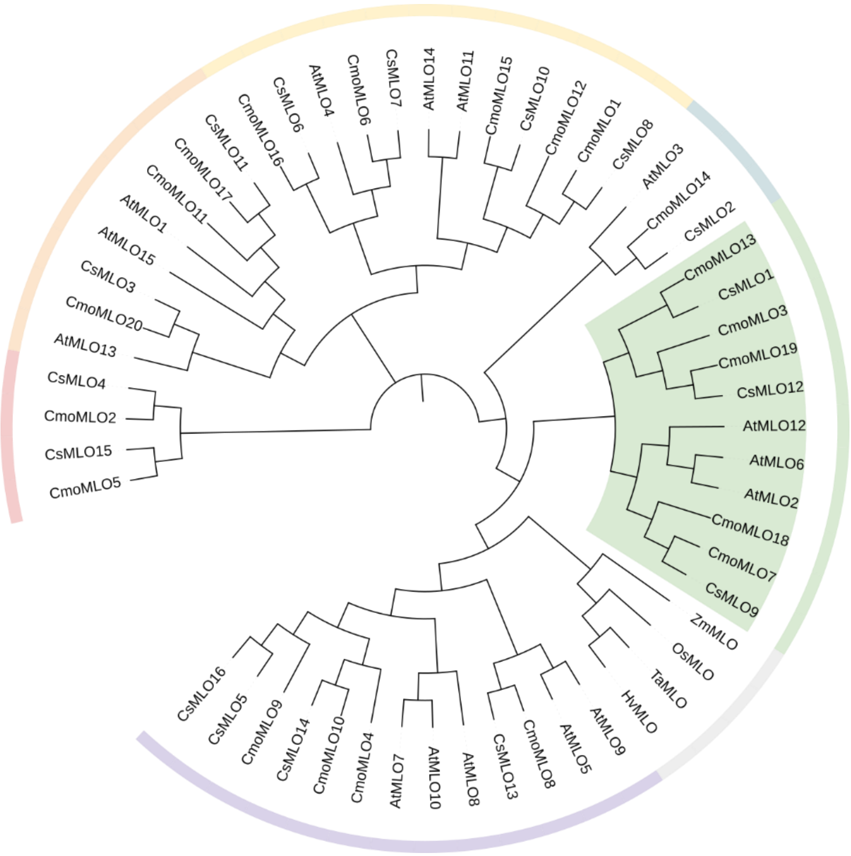

Figure S4: Phylogenetic tree of all MLO-like proteins from Arabidopsis (AtMLO1-15), cucumber (CsMLO1-16), *C. moschata* (CmMLO1-20), and functional MLO genes in four monocotyledonous crops (barley/*HvMLO*, wheat/*TaMLO*, rice/*OsMLO*, and maize/*ZmMLO*). The phylogenetic tree was constructed using MEGA 7.0.26 with the Maximum Likelihood method and 1000 bootstrap replications. The branch highlighted in light green represents the proteins from clade V.

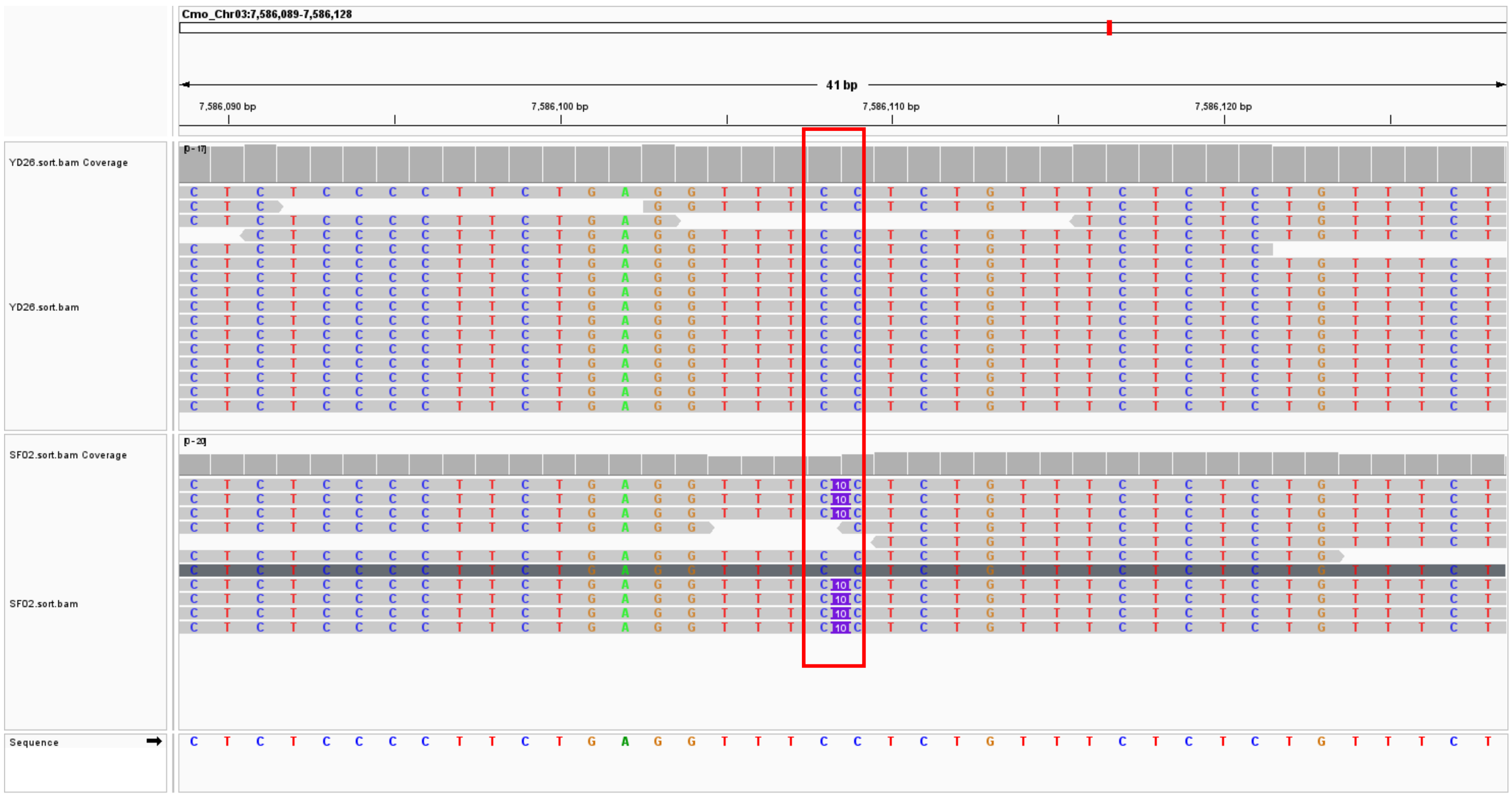
Cmo_Chr03:7,586,089-7,586,128
41 bp
7,586,090 bp
7,586,100 bp
7,586,110 bp
7,586,120 bp
YD26.sort.bam Coverage
YD26.sort.bam
SF02.sort.bam Coverage
SF02.sort.bam
Sequence

A

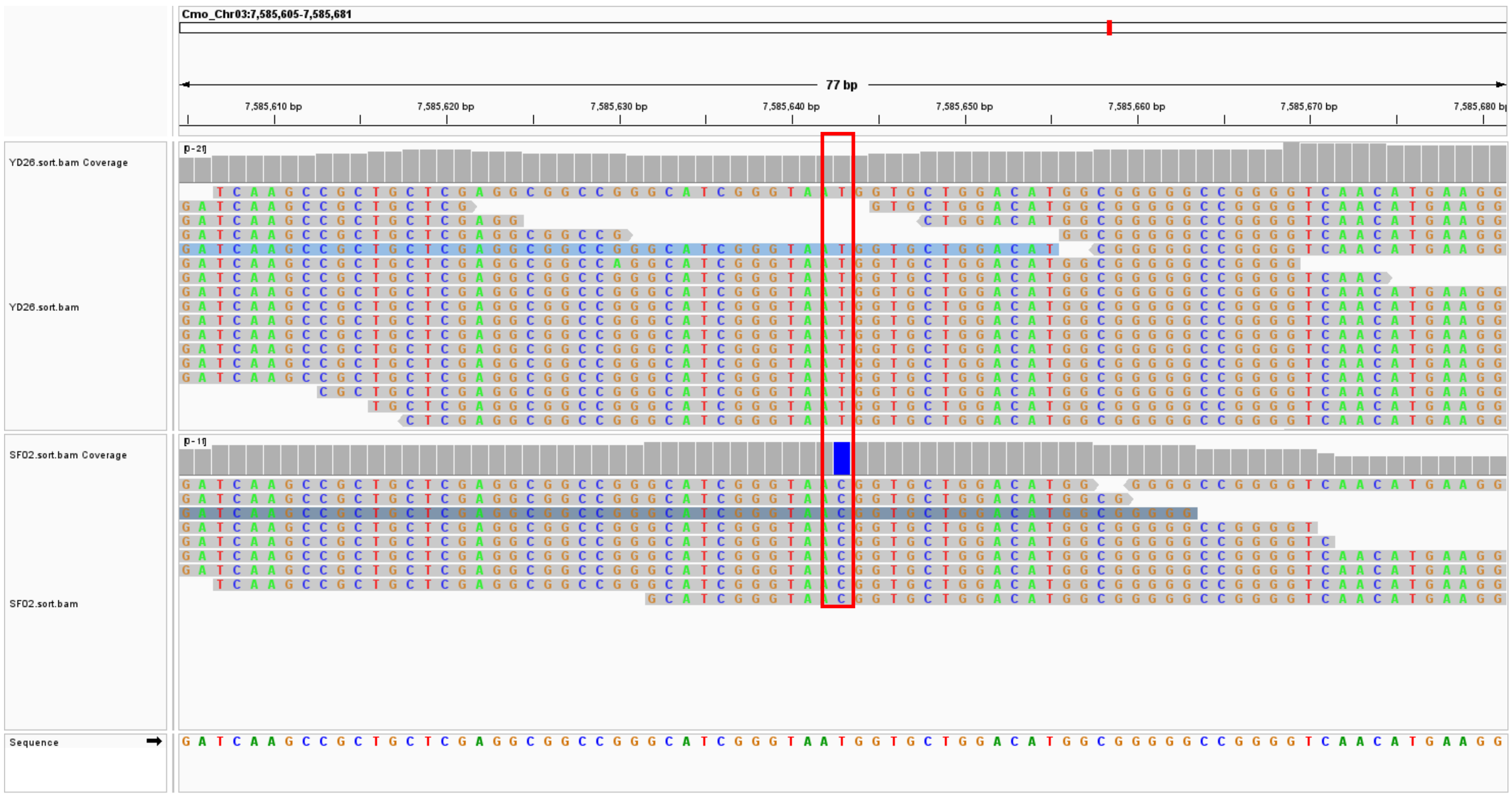
Cmo_Chr03:7,585,605-7,585,681
77 bp
7,585,610 bp
7,585,620 bp
7,585,630 bp
7,585,640 bp
7,585,650 bp
7,585,660 bp
7,585,670 bp
YD26.sort.bam Coverage
YD26.sort.bam
SF02.sort.bam Coverage
SF02.sort.bam
Sequence

B

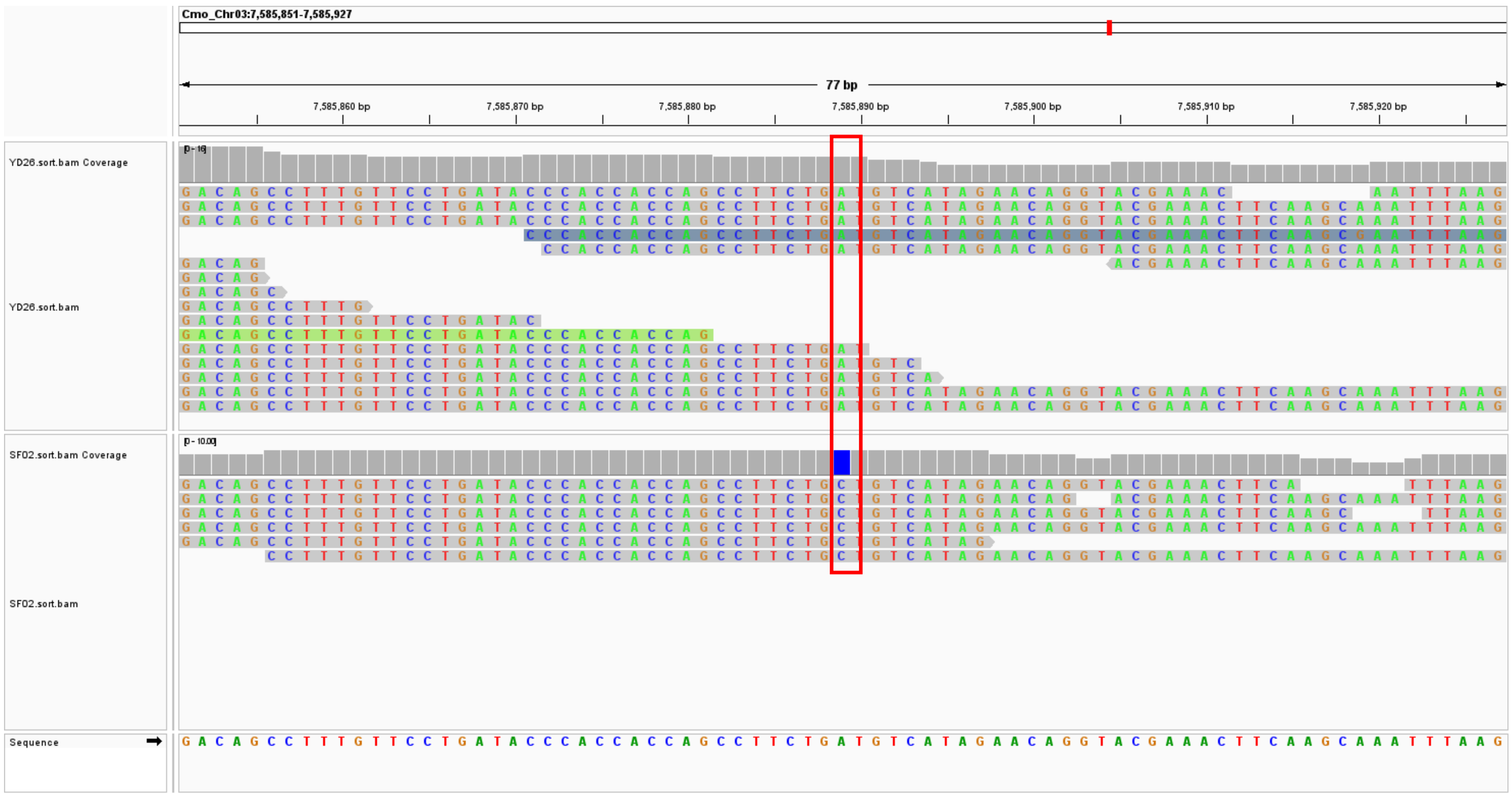
Cmo_Chr03:7,585,851-7,585,927
77 bp
7,585,860 bp
7,585,870 bp
7,585,880 bp
7,585,890 bp
7,585,900 bp
7,585,910 bp
7,585,920 bp
YD26.sort.bam Coverage
YD26.sort.bam
SF02.sort.bam Coverage
SF02.sort.bam
Sequence

C

Figure S5 Reads for genotypes YD26 and SF02 in the region of the *SABP2-like* gene CmoCh03G010100. (A) a 11bp insertion (C > CCTCTGTTTCT) at the position 7,586,108 on chromosome 3 of SF02. (B) the non-synonymous SNP (T > C) at the position 7,585,643 on chromosome 3 of SF02. (C) the non-synonymous SNP (A > C) at the position 7,585,889 on chromosome 3 of SF02.

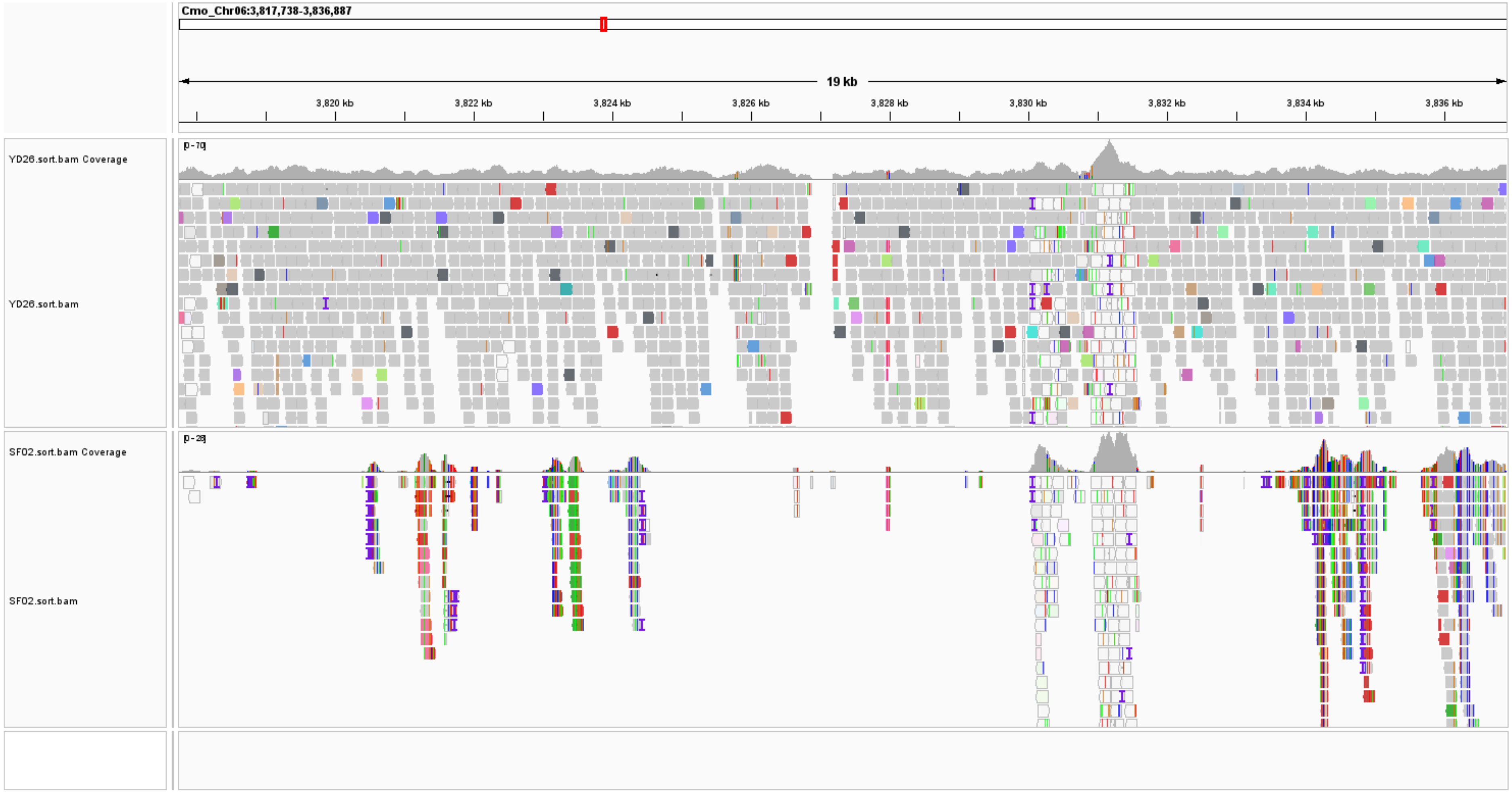

Figure S6: Visualization of sequencing reads for genotypes YD26 and SF02 in the region on Chromosome 6, spanning CmoCh06: 3,817,738 ~ 3,836,887.

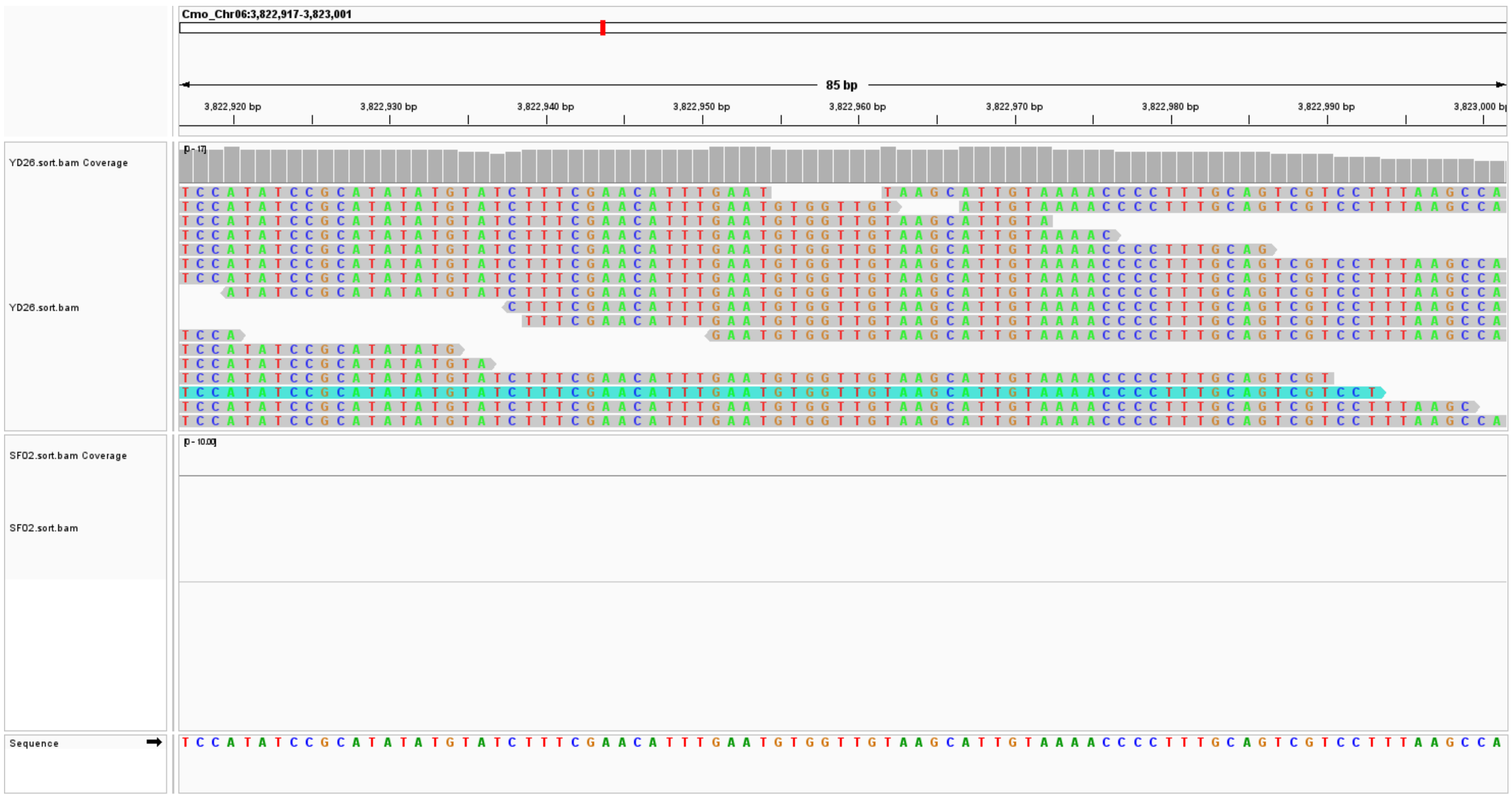
Cmo_Chr06:3,822,917-3,823,001
85 bp
YD26.sort.bam Coverage
YD26.sort.bam
SF02.sort.bam Coverage
SF02.sort.bam
Sequence

A

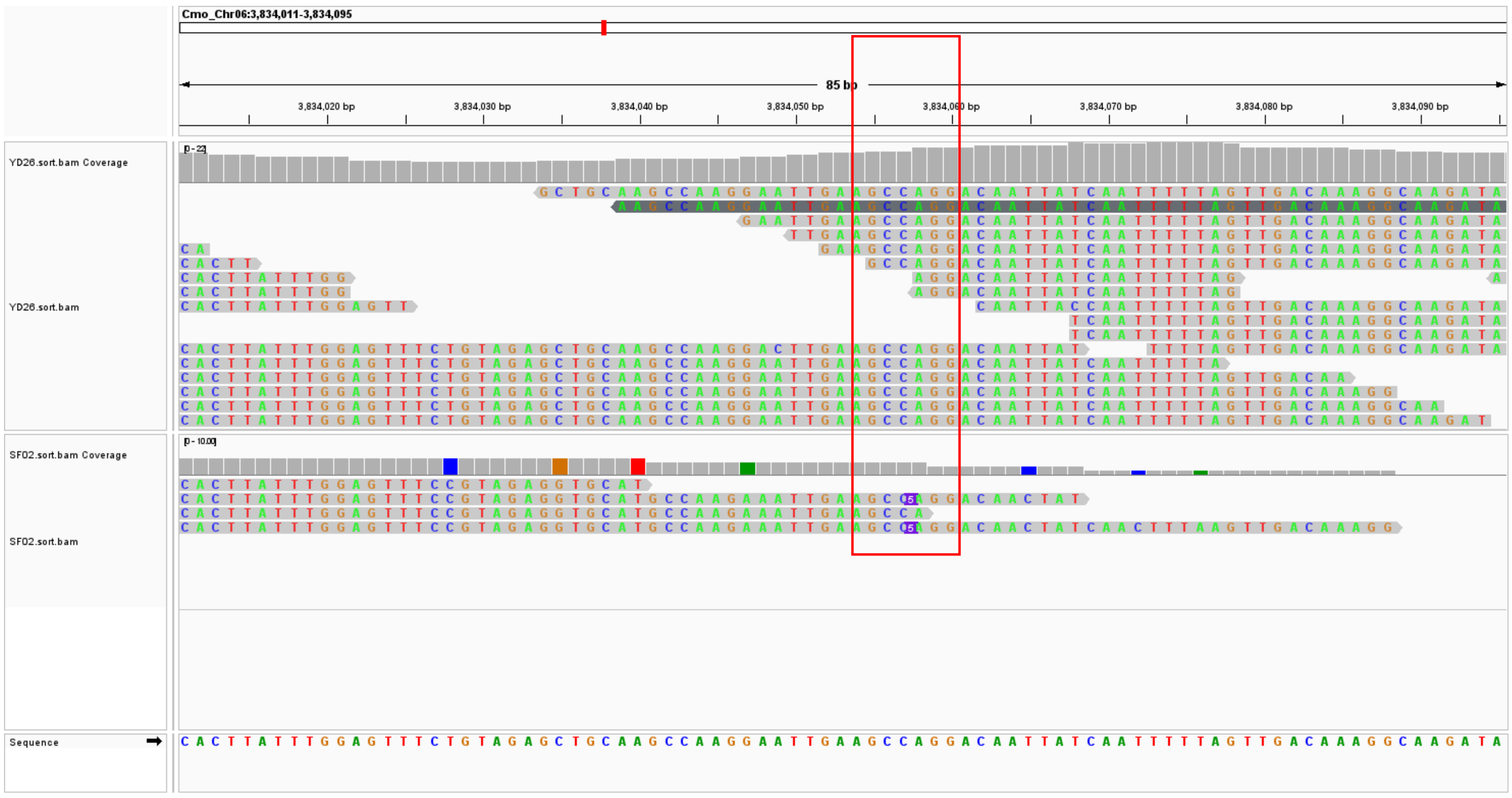
Cmo_Chr06:3,834,011-3,834,095
85 bp
YD26.sort.bam Coverage
YD26.sort.bam
SF02.sort.bam Coverage
SF02.sort.bam
Sequence

B

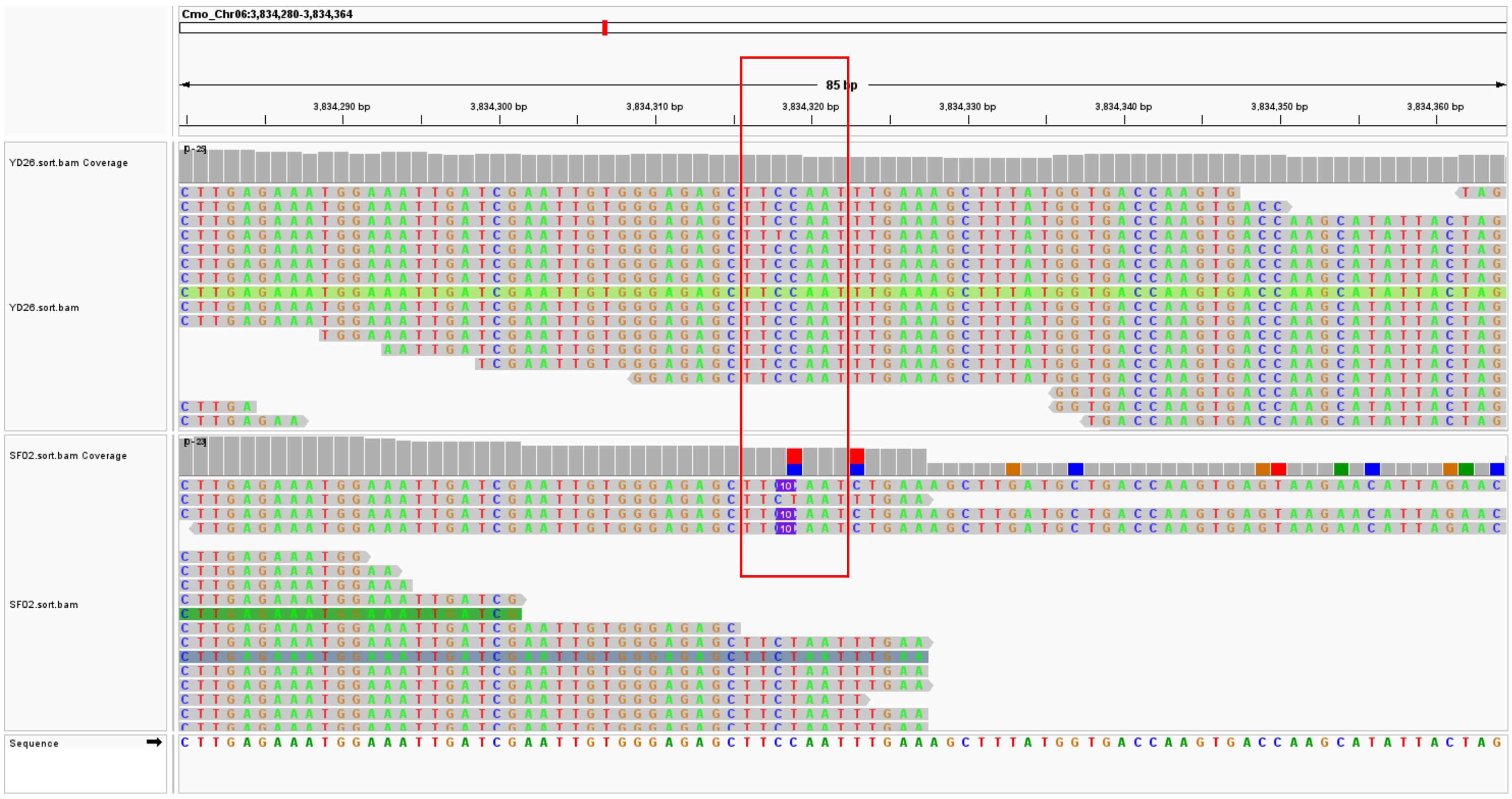
Cmo_Chr06:3,834,280-3,834,364
85 bp
YD26.sort.bam Coverage
YD26.sort.bam
SF02.sort.bam Coverage
SF02.sort.bam
Sequence

C

Figure S7 Reads for genotypes YD26 and SF02 in the region of the R gene cluster on chromosome 6. (A) codon change plus codon deletion at the position 3,822,992 on chromosome 6, CmoCh06G007400 (ttaaag>ttg). (B) frame shift at the position 3,834,057 on chromosome 6, CmoCh06G007410 (cct/cctAAATT). (C) frame shift at the position 3,834,318 on chromosome 6, CmoCh06G007420 (tgg/tgCTTCAAATTAg).
